# The effect of adding visual summaries to data visualizations on patient judgments of hypertension control

**DOI:** 10.1093/jamiaopen/ooag034

**Published:** 2026-03-24

**Authors:** Victoria A Shaffer, Sean Duan, Pete Weiger, Michelle Bobo, Shannon M Canfield, Abigail Rolbiecki, Talile M Geleto, William Martinez, Richelle Koopman, David Dorr

**Affiliations:** Department of Psychological Sciences, University of Missouri, Columbia, MO, United States; Department of Psychological Sciences, University of Missouri, Columbia, MO, United States; Humber River Health, University of Toronto, Toronto, Canada; Care Management Plus, Oregon Health & Science University, Portland, OR, United States; Department of Family and Community Medicine, University of Missouri, Columbia, MO, United States; Department of Family Medicine, University of Colorado, Aurora, CO, United States; University of Washington, Seattle, WA, United States; Department of Medicine, Vanderbilt University, Nashville, TN, United States; Department of Family and Community Medicine, University of Missouri, Columbia, MO, United States; Division of General Internal Medicine and Geriatrics, Oregon Health & Science University, Portland, OR, United States

## Abstract

**Objectives:**

To test the impact of visual summaries of blood pressure (BP) data (eg, stoplight and gradient displays), within the context of a patient-facing digital application connected to the EHR, on patient judgments about hypertension control.

**Materials and Methods:**

Participants (N = 117; Internet sample of patients with hypertension) viewed graphs depicting BP data for fictitious patients. For each graph, participants rated perceived hypertension control, risk of heart attack and stroke, urgency, worry, and perceived understanding of health implications on a 0-100 slider bar and indicated the preferred action to take in response this BP data (eg, talk to doctor at next appointment, go to hospital immediately). Using a within-subjects design, all participants evaluated 12 graphs with data that varied in systolic BP mean (controlled or uncontrolled) and standard deviation (moderate or high) and included three different types of visual summaries: (1) control (average BP only), (2) stoplight, (3) gradient. Participants also completed the Graph Literacy-Short Form and the Electronic Health Literacy Scales (eHEALS).

**Results:**

Measures of perceived risk of heart attack and stroke, urgency, and worry were significantly greater and perceived hypertension control was significantly lower for cases where hypertension was uncontrolled *P* < 0.05. However, there were no significant differences between visual summary methods on the primary outcomes. Graph literacy and electronic health literacy were globally related to judgments of hypertension control but did not interact with any of the study factors.

**Discussion and Conclusion:**

The verbal summary, stoplight, and gradient displays performed similarly despite the addition of more precise risk information.

## Introduction

Hypertension, characterized by persistently elevated blood pressure, affects nearly 1.28 billion adults globally.^1^ Despite advances in pharmacological treatments, managing hypertension remains a substantial issue, due to the asymptomatic nature of the disease and poor adherence to antihypertensive medications, and continues to be a significant risk factor for severe health complications including cardiovascular disease, stroke, and chronic renal disease.^2^ Home blood pressure measurement is often recommended as it can provide a more accurate assessment of control and facilitate better self-management behaviors.^3^ However, patient engagement in home blood pressure measurement has been limited because it is a complex and multifaceted process that involves individualized treatment plans and continuous monitoring, along with the need for patient adherence to medication and lifestyle changes.^4^

To address the challenges of hypertension control through home blood pressure management, our research team developed the Collaboration Oriented Approach for Controlling High blood pressure (COACH), which is a Fast Healthcare Interoperability Resource based digital application connected to the Electronic Health Record. COACH is a patient-facing application that generates guideline-supported messaging and data visualizations using clinician- and patient-supplied data to help patients control their blood pressure. The main page of the application displays a prominent graph of the patient’s recent blood pressure history, and COACH utilizes clinical decision support algorithms to provide treatment suggestions based on the patient’s medical record, including lifestyle modifications and medication-based therapies. COACH also alerts the patients to dangerously high or low readings and notifies them to contact their care team. While COACH is accessible to both patients and clinicians, it is designed to support patient home management of hypertension, providing patients with the tools needed to evaluate their progress and the ability to connect with their care team when there are areas of concern.

Early versions of the COACH data visualization tool were developed through a rapid prototyping process that used feedback from patients and clinicians to iteratively update a series of candidate visualizations.^5^^,^^6^ The resulting display was further refined after a set of vignette-based studies identifying factors that influence patient and physician judgments of hypertension control.^7–10^ This work led to a final prototype featuring an enhanced graph employing a smoothing line generated by a locally weighted smoothing (LOWESS) algorithm that presents a mean value for a specific interval size. Data visualization with the smoothing function directed patients and physicians to clinically meaningful elements of the display while minimizing the impact of visual noise (eg, variability in measurements and outliers). These enhanced displays resulted in judgments about hypertension control that were more consistent with clinical guidelines than judgments using data tables and graphs of only the raw data.^8^^,^^10^

While the data visualization tool developed for COACH globally improved judgments about hypertension control in patient samples, the enhanced data visualization had the greatest impact on patients with high graph literacy.^7^ Patients with the lowest levels of graph literacy were not able to accurately distinguish between cases of controlled and uncontrolled hypertension even with the enhanced data display. Because health information technologies, like COACH, can encourage patients to take an active role in monitoring and managing their symptoms and become more engaged in decision making about their hypertension care, it is important to find methods of communicating about complex quantitative information that allow patients with lower numeracy and health literacy levels to effectively engage with digital health interventions.^11^^,^^12^

To increase the accessibility and usability of the COACH application, we examined the addition of visual summaries to data visualization in COACH to increase comprehension about blood pressure data and its implication for a patient’s clinical care. Visual summary techniques, such as stoplight and gradient displays, can improve communication of complex data and motivate patients to participate in self-management activities via the strategic use of color and symbolism that provides evaluative labels (ie, “out of range”) and context for quantitative information.^13^ Visual summaries have been developed for other areas of health communication, including viewing test results via patient portals of electronic health record (EHR) systems, where understanding medical test results often requires patients to interpret complex numerical data, which can be overwhelming, particularly for individuals with limited health literacy.^12^

Visual displays of health information are most effective when pictures are used to illustrate key messages (eg, your blood pressure is outside the goal range), text is simplified and consistent with the message conveyed by the visualization, and unnecessary details are omitted.^13^ The most helpful visualizations increase comprehension by providing “a clear take away message”, give meaning to results by contextualizing quantitative information, and provide clear thresholds for action.^13^^,^^14^ The stoplight display is a frequently used visualization that draws on the familiar analogy of a traffic light enabling sense making by providing context for interpretation of quantitative health information This method was designed to engage patients with low health literacy in research, employing the colors of traffic light symbols (green, yellow, red), which are nearly universally understood, to convey gist level information.^15^ This method can be used to increase comprehension of quantitative health information, such as laboratory test results, by taking a numerical value that is continuous in nature (eg, blood pressure, a1c, cholesterol) and categorizing scores as either green (within a recommended range), yellow (borderline), or red (exceeds a critical value).

The use of a stoplight or traffic light display has been beneficial in home management of patients with heart failure, improving patient knowledge and increasing self-care behaviors.^16^ The stoplight display has also been applied to education about nutrition and resulted in increased selection and consumption of health foods.^17^ The effects of traffic light labels have also been shown to have a sustained longitudinal impact. Over a two-year period, traffic light displays resulted in the decrease in purchasing of ‘red light’ food and drink items and an increase in purchasing of ‘green light’ food and drink items in a large hospital cafeteria.^18^

While stoplight displays have been effective in improving comprehension of health information, patients have indicated that these displays are not preferred because they are overly simplified, valuing instead infographics that they perceived to have more information.^13^ Other methods of visualization, such as gradient displays, use a similar method of contextualizing quantitative health information but include more precise communication about risk information.^19^^,^^20^ These displays are similar to the traffic light method in that the interpretation of numerical values ranges across color variations (eg, green-yellow-red spectrum). However, instead of truncating the scale into three discrete categories, the visual transition between the categories is smoothed preserving the continuous nature of the underlying relationship of the numerical value to its corresponding risk. The value of this approach is that it keeps patients from interpreting all out of range values similarly. Values that are only slightly outside the goal range should be met with less worry than values substantially outside of the goal range, even though these values would be interpreted in the same way with the stoplight method.

Gradient displays have been used in the communication of laboratory tests within the EHR.^12^^,^^19–21^ When compared with lab test results presented in tables, the use of gradient visual displays to present results that are near-normal, or slightly outside the goal range, reduce the perceived urgency of patients and decreased their desire to contact their health provider immediately.^19^ Thus, the gradient displays increase patient sensitivity to the values of test results, enabling them to differentiate between values that are slightly outside the goal range and those that are significantly outside the goal range. Further, the inclusion of a harm anchor or other risk-related reference points on a gradient scale to demarcate regions where substantial patient risk may occur has also been shown to increase the interpretability of laboratory test results for patients.^20^

This study was designed to evaluate the effectiveness of stoplight and gradient displays, within the context of the COACH digital application, addressing two specific research questions: (1) Do visual displays impact patient judgments about hypertension control? (RQ1) (2) Does more precise risk information, present in the gradient display, facilitate more appropriate responses to the data (eg, action in the case of clinically urgent values or inaction in the case of values that are only slightly outside the goal range) (RQ2)? We compared a standard version of the COACH application without a visual summary of the blood pressure data to versions featuring the two types of visual summary displays. Both the stoplight and gradient displays provided evaluative labels for quantitative information (ie, identifying blood pressure values outside the target range), but the two differed in the precision of risk information provided. We examined the impact of visual summaries on patient judgments about hypertension control, including perceived need for medication change, risk of heart attack and stroke, comprehension, and urgency. These outcomes were chosen because they represent real world judgments made by patients when using home blood pressure monitoring tools. Any actions taken as a result of such a tool would be dependent upon a patient’s judgment about their hypertension control and would be mediated by their beliefs about how well they understand the information presented and what it means for their heath.

In considering RQ1, we hypothesized that judgments about hypertension control would differ by visual display, with the stoplight and gradient displays resulting in greater discrimination between cases of controlled and uncontrolled hypertension (H1a). Further, we anticipated that the effect of visual display would be greater for patients with lower levels of graph literacy (H1b). With respect to RQ2, we did not have specific a priori hypotheses about the superiority of the gradient display to the stoplight display. While there is a reasonable theoretical rationale for predicting that more precise risk information can result in more clinically appropriate responses to the data, there is other research on data visualization which suggests that “less is more” when it comes to visualization of health information (eg,^22^).

## Method

### Study design

An internet sample of patients with hypertension reviewed several brief vignettes describing a fictitious patient; each vignette included a graph of the patient’s blood pressure data. This work was reviewed and approved by the Institutional Review Board affiliated with the first author’s institution. All participants were recruited by Prolific, a survey company that maintains an opt-in Internet panel that participates in survey research in exchange for small incentives. Participants with hypertension were identified via a single self-reported measure: ‘Has your doctor ever diagnosed you with hypertension, also known as high blood pressure?’; similar self-report items have been used to identify patients with hypertension in other epidemiologic studies.^23–25^ This method was similar to other published vignette-based studies that used samples of patients with hypertension.^7–9^

Each vignette described a patient being treated for hypertension and included a visualization of the patient’s blood pressure data over the past 2 months. There were 12 vignettes that systematically varied in three attributes: (1) mean systolic blood pressure (SBP: controlled or uncontrolled); (2) blood pressure standard deviation (SD: moderate or high); (3) type of visual summary (control, stoplight, or gradient).

**Figure 1.**
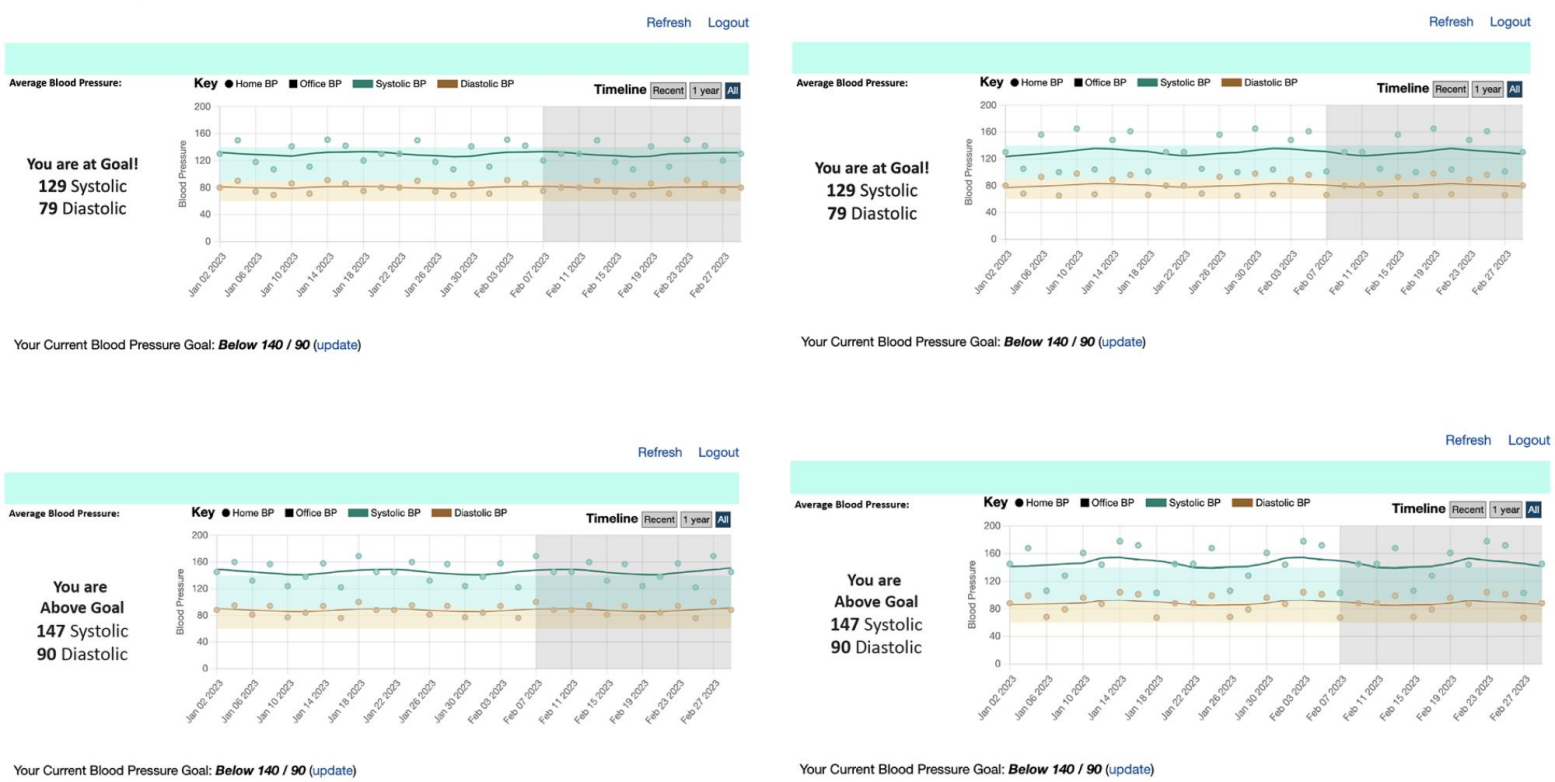
The four clinical cases represented in the vignettes shown in the control condition from top left to bottom right: (1) controlled hypertension, moderate variability; (2) controlled hypertension, high variability; (3) uncontrolled hypertension, moderate variability; and (4) uncontrolled hypertension, high variability.

Half of the vignettes illustrated cases of controlled hypertension (SBP Mean of 129 mmHg), while the other half of the vignettes illustrated cases of uncontrolled hypertension (SBP Mean of 147 mmHg). Mean SBP values were chosen to represent sample clinical cases that depicted controlled or uncontrolled hypertension according to the 2014 Hypertension Management Guideline.^26^ Further, half of the vignettes depicted cases of moderate variability (SBP SD = 15), and the other half of the vignettes were cases of high variability (SBP SD = 25). These standard deviations were chosen to represent moderate and large mean variability according to published SBP values.^27^ The combination of these two factors resulted in four unique clinical cases: (1) controlled hypertension with moderate variability, (2) controlled hypertension with high variability, (3) uncontrolled hypertension with moderate variability, and (4) uncontrolled hypertension with high variability. See Figure 1 for vignette details and examples of the four clinical cases shown in the COACH display.

**Figure 2.**
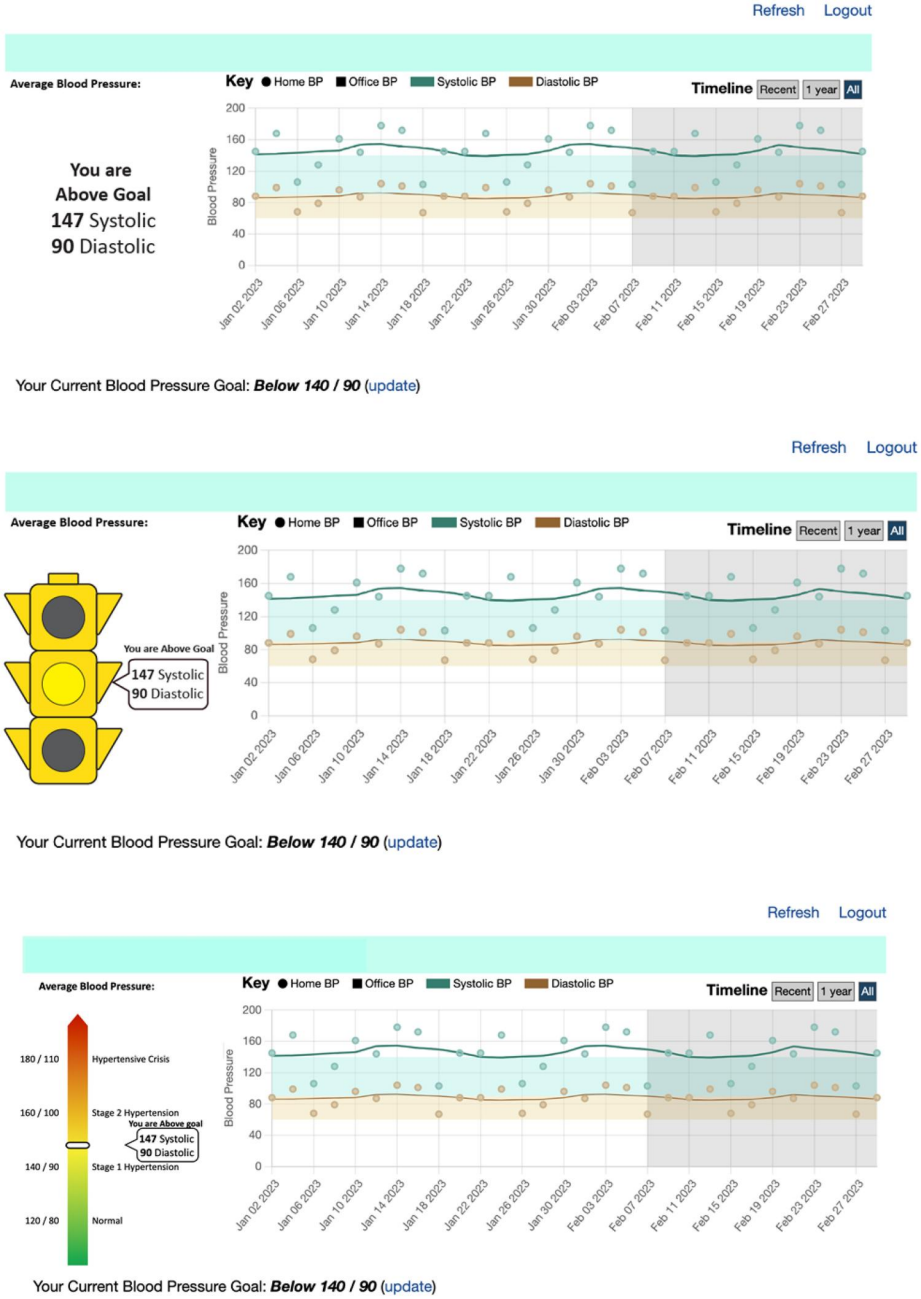
The three visual summary conditions represented in the vignettes from top to bottom are: 1) the control condition, which only provides the average blood value and indicates whether the patient was at or above their blood pressure goal; 2) the stoplight condition, which includes the average blood pressure and a stoplight to illustrate whether the patient's blood pressure is considered in the standard range, elevated, or a hypertensive crisis; 3) the gradient condition, which includes the average blood pressure and a red-yellow-green continuum that identifies 5 evaluative categories: standard range, elevated, stage 1 hypertension, stage 2 hypertension, and hypertensive crisis.

The vignettes also differed in the use of a visual summary. Each of the four clinical cases was depicted as a vignette with a verbal summary only, a stoplight display, and a gradient display. The verbal summary served as the control condition, with no visual summarization; the text above the average blood pressure value indicated whether the patient in the vignette was at or above their blood pressure goal. The stoplight display identified whether the patient depicted in the vignette was at or above their blood pressure goal using the visual analogy of the stop light. We categorized blood pressure readings into three color-coded zones that correlate with controlled, borderline, and critical blood pressure levels, respectively. Patients in the “green” zone were considered to have blood pressure within a safe range. Readings in the “yellow” signaled that patients had elevated or “above normal” values that corresponded to a diagnosis of hypertension according to the 2014 Hypertension Management Guideline.^28^ “Red” zones indicated values that are considered to be a hypertensive crisis. In contrast, the gradient display did not have discrete categories identified but did include five evaluative labels on the continuum: Standard range, elevated, hypertension stage 1, hypertension stage 2, and hypertensive crisis. The gradient display provided the most precise information for interpreting the average blood pressure value, by presenting each patient case along a continuum of diagnostic categories. See Figure 2 for examples of the visual summary conditions. The study used a 2 (SBP Mean) × 2 (SBP SD) × 3 (visual summary type) within-subjects design, where all participants reviewed all 12 vignettes, with order of presentation randomized, and provided judgments about the degree of hypertension control for every vignette.

### Outcomes

Primary outcomes were: (1) perceived hypertension control, (2) perceived risk of heart attack and stroke, (3) perceived urgency, (4) worry, and (5) perceived understanding of health implications. These outcomes were measured using a slider bar; see Table 1 for item working and scale ranges. These items were used in prior work to measure judgments of hypertension control.^7–10^

**Table 1.** Perceived hypertension control, heart attack and stroke risk, urgency, worry and perceived understanding of health implications by vignette, M(SD)

Items	Verbal summary	Stoplight	Gradient
*This patient’s blood pressure is well controlled.* (0 ‘Strongly disagree’—100 ‘Strongly agree’)			
Controlled hypertension	73.15 (27.86)	74.01 (27.38)	71.61(28.11)
Uncontrolled hypertension	36.29 (23.25)	35.77 (22.35)	36.06 (23.43)
*How likely is this patient to have a heart attack or stroke in the next 10 years?* (1 ‘Extremely unlikely’—10 ‘Extremely likely’)			
Controlled hypertension	3.14 (2.00)	3.04 (2.00)	3.22 (2.05)
Uncontrolled hypertension	5.71 (2.03)	5.79 (1.97)	5.73 (2.11)
How urgent is this? (0 ‘Not at all urgent’—6 ‘Extremely urgent’)			
Controlled hypertension	1.31 (1.53)	1.22 (1.43)	1.35 (1.44)
Uncontrolled hypertension	3.23 (1.42)	3.20 (1.38)	3.23 (1.43)
How worried would you be? (0 ‘Not at all worried’—6 ‘Extremely worried’)			
Controlled hypertension	1.25 (1.53)	1.26 (1.51)	1.43 (1.55)
Uncontrolled hypertension	3.25 (1.48)	3.27 (1.45)	3.20 (1.51)
How well do you understand what this blood pressure data means for this patient’s health? (0 ‘Not at all’—6 ‘Completely’)			
Controlled hypertension	4.94 (1.17)	4.99 (1.08)	4.95 (1.25)
Uncontrolled hypertension	4.71 (1.18)	4.77 (1.08)	4.74 (1.17)

We also measured the preferred action to take in response to the BP data shown in each of the 12 vignettes. Participants were asked to select a category of action they would recommend from the following options: (1) Do nothing; (2) Talk to the doctor about their blood pressure at the next regularly scheduled appointment; (3) Ask to see the doctor at the first available appointment; (4) Go to a hospital or doctor’s office tomorrow; (5) Go to a hospital as soon as they were free today; and (6) Go to a hospital immediately. This item and its response categories were adapted from prior work on the use of gradient visual summaries to distinguish between urgent and non-urgent laboratory test results.^19^^,^^20^ For the purposes of interpreting these results, we combined the response categories into three groups: (1) No immediate action, (2) Appointment soon, and (3) Hospital today.

Participants also completed the Graph Literacy-Short Form (GLSF)—a 4-item scale measuring the ability to understand information presented in a graph,^28^—and the 8-item Electronic Health Literacy Scale (eHEALS) developed by Norman and Skinner.^29^ Each of the four items in the GLSF is an objective measure where participant responses are scored as correct or incorrect. The composite score on this scale represents the total number of correct responses. eHEALS is designed to measure participants' perceived skill in using health information technology (eg, I feel confident in using information from the Internet to make health decisions; 1 = Strongly Disagree, 5 = Strongly Agree). The composite score on this scale is an average of responses to the 8 items. Additionally, participants provided demographic information about age, gender, ethnicity, race, income, and education and answered two items about the frequency of monitoring and graphing their own blood pressure at home.

### Power and statistical analyses

We planned to recruit 100 participants who had previously been diagnosed with hypertension. This sample size was determined using G-Power,^30^^,^^31^ where we identified the sample needed to achieve a power of 90%, at a significance level of 0.05 where we assumed a small effect size (Cohen’s *f* = 0.10), a correlation of 0.50 between repeated measures, and continuous outcomes.

We conducted a series of repeated-measures analysis of variance (ANOVA) models with each of the primary outcome variables (perceived hypertension control, perceived heart attack and stroke risk, worry, perceived urgency, and perceived understanding of health implications). To examine research questions about the impact of visual summaries, we included type of visual summary (control, stoplight, or gradient), SBP mean (controlled or uncontrolled), and SD (moderate or high) as fixed effects in these models, examining their main effects as well as their two and three-way interactions. Family-wise error was constrained in the ANOVA models using the Bonferroni correction method. For each of the models, the assumption of normality was examined using qq plots. Given that ANOVA is robust to violations of normality, particularly with skewed distributions and distributions with thinner tails, we focused on the detection of distributions with thicker tails. Sphericity was determined using Mauchley’s test; where appropriate Geisser-Greenhouse $\hat{\varepsilon }$ adjusted *p*-values are reported.

To examine research questions about the moderating role of electronic health literacy and graph literacy, we used a series of mixed-effects models to examine the impact of these individual differences on the relationship between the experimental fixed effects (type of visual summary, SBP mean, SD) and the five primary outcomes. We tested models with both random and fixed intercepts and slopes with each outcome as the level 1 variable and participant as the level 2 variable, with patient scores on eEHALS and the GLSF included as level 2 predictors. The final models were selected using AIC (Akaike Information Criteria) and BIC (Bayesian Information Criteria) and are described below. Analyses were conducted in R version 4.4.1,^32^ and linear mixed models were fit using the nlme package in R.^33^  *P* < 0.05 was considered significant for all results.

## Results

### Participants

A total of 117 patients with hypertension participated in this study. Approximately half of the participants were male (52.10%), and the majority of participants identified as White (82.10%). Participants ranged in age from 40 to 81, with a mean age of 54.97 years (SD = 9.31). Because Internet samples tend to be more educated than the general population, we oversampled participants with lower levels education. Of the sample, 51% had a high school degree or some college, 25% had a bachelor’s degree, and 24% had an advanced degree. Many participants in this sample were familiar with home blood pressure monitoring, with more than half of the sample taking their blood pressure at home daily or weekly. See Table 2 for complete participant characteristics.

**Table 2.** Participant characteristics

Demographics		*N* = 117
Age; Mean (SD)		54.97 (9.31)
Gender; *N* (%)	Female	61 (52.10%)
	Male	56 (47.90%)
Latinx; N (%)		4 (3.41%)
Graph Literacy Scale, Short Form; M (SD)		2.28 (0.87)
Graph Literacy Scale—Number of correct items; N (%)		
0		2 (1.71%)
1		19 (16.24%)
2		47 (40.17%)
3		42 (35.90%)
4		7 (5.98%)
Electronic Health Literacy Scale (eHEALS); M (SD)		4.28 (0.65)
Race; N (%)	American Indian or Alaskan Native	0
	Asian	2 (1.91%)
	Native Hawaiian or Other Pacific Islander	0
	Black or African American	16 (13.68%)
	White	96 (82.05%)
	Other	0
	Asian, Native Hawaiian or Other Pacific Islander	1 (0.85%)
	American Indian or Alaskan Native, Native Hawaiian or Other Pacific Islander	1 (0.85%)
	White, American Indian or Alaskan Native	1 (0.85%)
Education; *N* (%)	High school graduate or GED	12 (10.25%)
	Some college, no degree	20 (17.94%)
	Trade, technical, or vocational training	4 (3.41%)
	Associate’s degree	24 (20.51%)
	Bachelor’s Degree	29 (24.77%)
	Master’s degree	27 (23.08%)
	Doctoral Degree	1 (0.85%)
How often do you monitor your blood pressure at home?		
Never		14 (11.97%)
Annually		8 (6.84%)
Monthly		34 (29.06%)
Weekly		38 (32.48%)
Daily		23 (19.66%)
How often do you graph your home blood pressure measurements?		
Never		73 (62.39%)
Annually		7 (5.98%)
Monthly		11 (9.40%)
Weekly		21 (17.95%)
Daily		5 (4.27%)

### Primary outcomes

While there is no objectively correct level of worry or urgency a patient should experience if their blood pressure is outside of the goal range, effective decision support tools will allow patients to titrate their level of concern to the clinical context. That is, perceived hypertension control should be greater in cases where blood pressure is within the goal range, and measures of worry and perceived urgency should increase when blood pressure is outside the goal range. And that is precisely what is observed in this study. There was a significant effect of SBP mean on perceived hypertension control [*F*(1,116) = 260.43, *P ≤* .001], perceived risk of heart attack and stroke [*F*(1,116) = 262.78, *P* < 0.001], perceived urgency [*F*(1,116) = 254.265, *P* < 0.001], and worry [*F*(1, 116) = 232.00, *P* < 0.001]. Indicating that vignettes depicting uncontrolled hypertension were given higher ratings of perceived risk, urgency, and worry, along with lower ratings of perceived hypertension control. However, perceived understanding of health implications did not differ by SBP mean. That is, there was no difference in the ratings of perceived understanding of health implications for vignettes depicting controlled and uncontrolled hypertension, with participants rating their comprehension of the blood pressure data as high in both clinical contexts. Means and standard deviations for all primary outcomes are reported in Table 1.

**Figure 3.**
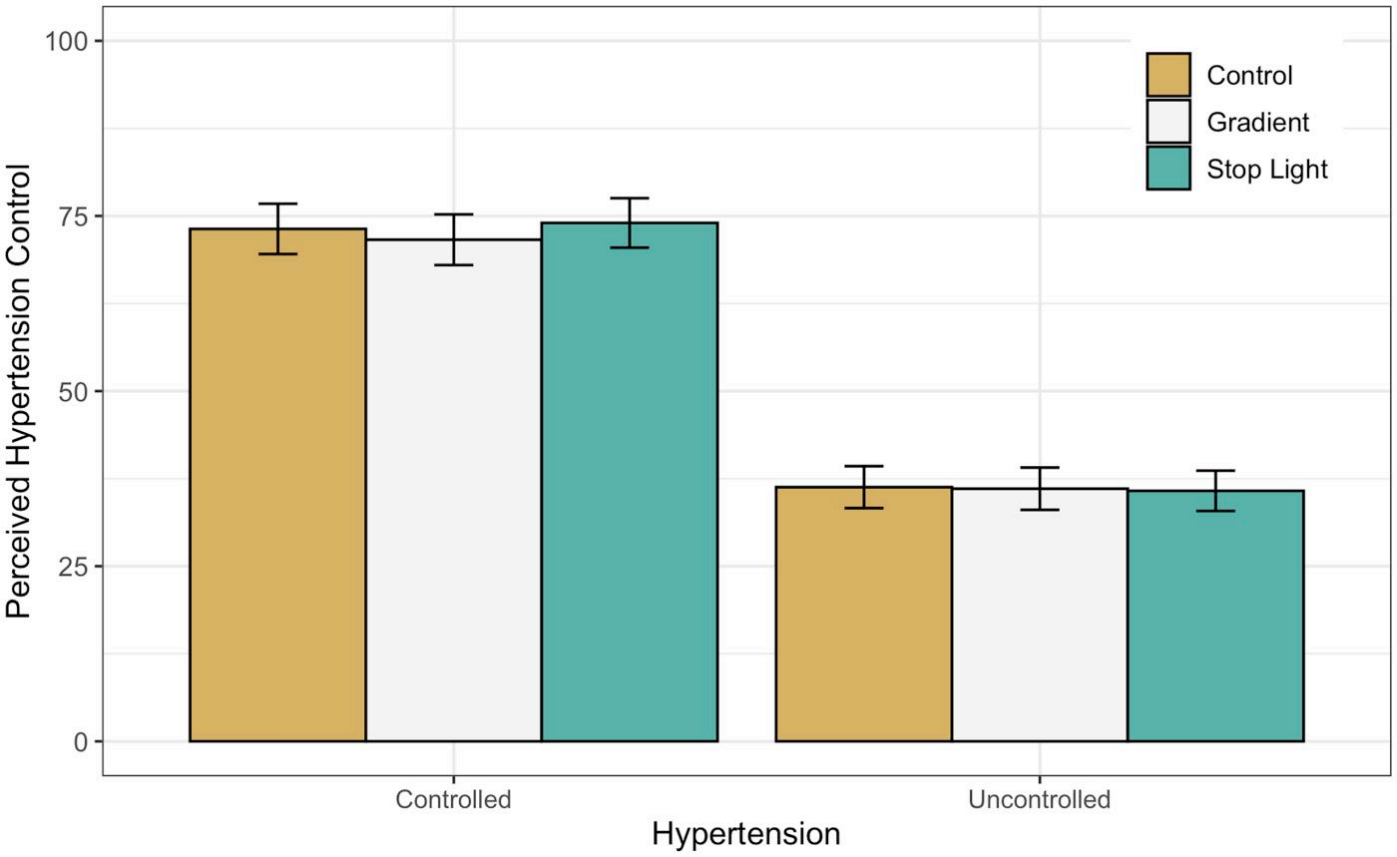
Perceived hypertension control was greater for cases with controlled hypertension compared to cases with uncontrolled hypertension, but there were no differences in ratings of perceived hypertension control between the control, gradient, and stoplight conditions.

To examine the impact of the use of visual summaries, we looked at the main effect of this factor as well as interactions with this factor and other experimental fixed effects in this study (SBP mean and SD) in models for each of the primary outcomes. We found no differential impact of visual summary method on perceptions of hypertension control (see Figure 3), risk, urgency, worry, or comprehension, *P* > 0.05. In each case, responses to the same clinical case presented with the control, stoplight, and gradient displays were similar for all primary outcomes. There were also no significant interactions between the visual summary factor and the other fixed effects in the study.

### Recommended actions in response to clinical vignettes

Responses to the item measuring the preferred action to take in response to the data are presented in Table 3. Like the primary outcomes, we would expect recommended actions to differ for vignettes where hypertension was controlled versus uncontrolled. Consistent with that hypothesis, participants were most likely to endorse ‘no immediate action’ (88.6% of participants chose this response) when blood pressure was in the goal range than for the vignettes where blood pressure was outside the goal range (47.29% of participants chose this response). However, there were no differences in responses among the visual summary conditions (control, stoplight, and gradient).

**Table 3.** Behavioral intention by vignette, N(%)

	Verbal summary	Stoplight	Gradient
Controlled hypertension			
No immediate action	211 (90.17%)	208 (88.89%)	203 (86.75%)
Appointment soon	17 (7.26%)	24 (10.26%)	28 (11.97%)
Hospital today	6 (2.56%)	2 (0.85%)	3 (1.28%)
Uncontrolled hypertension			
No immediate action	111 (47.43%)	110 (47.01%)	111 (47.44%)
Appointment soon	113 (48.29%)	114 (48.72%)	111 (47.44%)
Hospital today	10 (4.27%)	10 (4.27%)	12 (5.13%)

### Effects of graph literacy and electronic health literacy

We hypothesized that visual summaries with greater precision of risk (ie, gradient display) would benefit patients with high graph literacy, whereas less precise risk categories (ie, stoplight display) would benefit patients with low graph literacy. To test this hypothesis, we examined interactions between visual summary type and patient scores on the Graph Literacy-Short Form and eHEALS. Descriptive statistics for the scales are reported in Table 2. The Cronbach’s alpha observed in this sample for eHEALS was 0.94. However, models with interaction terms did not improve the fit of the mixed-effects models. All final models selected using AIC and BIC criteria included only the simple effects of graph literacy and electronic health literacy but no interactions.

Graph Literacy-Short Form, which measures the ability to extract information from a graph, was a significant predictor of perceived hypertension control, *β* = −3.51, *t* (114) = −2.09, *p* = 0.04, where greater graph literacy was associated with lower perceptions of hypertension control in all vignettes. However, there were no other significant effects on perceptions of heart attack and stroke risk, urgency, worry, or perceived understanding of health implications.

eHEALS, which measures the ability to find health information using digital health services, was a significant predictor of perceived understanding of health implications across all vignettes, *β* = 0.78, *t* (114) = 6.65, *p <* 0.001. Participants with higher scores on eHEALS also reported higher levels of perceived understanding of health implications across all vignettes. There were no effects of eHEALS on judgments of hypertension control, risk of heart attack and stroke, urgency, and worry. In sum, graph literacy and electronic health literacy were related to some of the primary outcomes, but there was no differential impact of the visual summaries by electronic health literacy or graph literacy.

## Discussion

Achieving hypertension control can lower the risk of death, cardio- and cerebrovascular events, kidney disease, and cognitive decline. Home blood pressure measurement with self-management is a crucial component of achieving control, but many patients struggle to consistently and accurately measure their blood pressure and then take appropriate action. Digital health interventions that intend to foster patient engagement need to leverage principles from cognitive science to increase motivation and drive behavior change without compromising comprehension, causing excessive worry, or seeking unnecessary clinical care.

In this study, the addition of visual summaries via stoplight displays and gradients to increase interpretation of the graphical information did not impact perceptions of hypertension control, risk of heart attack and stroke, urgency, worry, or comprehension, leading us to conclude that simplified verbal summaries alone are effective in helping patients extract important gist information (ie, “I am above my goal”) from noisy personal health data. Even the basic verbal summary of the blood pressure graph provided sufficient information for participants with lower scores on the Graph Literacy-Short Form and eHEALS to accurately assess hypertension control. An effective display would help patients calibrate their actions with the severity of the data, and this study indicated that all three visual summary methods performed well on this metric.

Other studies have shown that human-based support, often from nurses or health coaches, can increase home blood pressure measurement and self-management, lowering systolic blood pressure by 3.2 mmHg on average.^34^ With the advent of increasingly complex systems, having effective visual displays to guide the patient’s attention and drive action may enable more efficient use of health professionals’ time while achieving similar outcomes. Coupled with new interactive tools such as chatbots or interactive voice recognition systems,^35^ these displays may be able to be personalized and drive precise behavior change while minimizing both worry and burden for people with hypertension. Although tools of this type have yet to be proven—still often demonstrating improvements in knowledge rather than key behaviors—combining our knowledge of key clinical gaps with theories of motivation and engagement promises new opportunities for better control and outcomes.^36^

There are limitations to this study that may constrain its generalizability including the use of vignettes as a substitute for “real” clinical cases and an online sample to make judgments about hypertension control. However, the vignettes allow for experimental control over noisy clinical data increasing internal validity, and the use of an online sample mimics the way in which the home blood pressure management systems are designed to work. While there were no clear advantages to any of the three visual summary methods is this study, there may be other contexts in which there is value to the additional information provided by the stoplight and gradient displays that are not captured by these specific experimental materials. Future work should focus on other clinical contexts and include additional patient characteristics that may impact the effective use of technology to monitor hypertension control at home.

More research is needed to have a clear understanding about how the precision of risk information can facilitate or harm communication about health information. In some contexts, more precise information may be critical to home health management. However, this work suggests that simplified summaries alone can effectively assist patients with extracting key take-aways from noisy personal health data.

## Contribution statement

All authors have made substantial contributions to the conception or design of the work and/or the acquisition, analysis, or interpretation of the data. All authors were involved in either drafting the work or reviewing it critically for important intellectual content. All authors have approved the final version of the article and have agreed to be accountable for all aspects of the work.

## Data Availability

The data are available in a repository and can be accessed via the following link: https://osf.io/v35ca/? view_only=18b7de70077440c8930ac1573d9b0e95.
